# 114. Prospective Trial of Passive Diversion Device to Reduce Blood Culture Contamination

**DOI:** 10.1093/ofid/ofab466.114

**Published:** 2021-12-04

**Authors:** Sami Arnaout, Richard T Ellison, Thomas C Greenough, Azalea Wedig, Michael J Mitchell, Lauren St John, Shannon Stock

**Affiliations:** 1 UMass Memorial Medical Center, Westborough, MA; 2 University of Massachusetts Medical School, Worcester, MA; 3 College Of The Holy Cross, Worcester, Massachusetts; 4 College of The Holy Cross, Worcester, Massachusetts

## Abstract

**Background:**

Blood culture contaminants can lead to inappropriate antibiotic use, prolonged length of stay, and additional hospital costs. Several devices have been developed to reduce the risk of blood culture contamination by diverting a portion of the initial blood sample from the blood culture bottle. We have assessed the effectiveness of one blood diversion device in a prospective trial performed at the two separate emergency departments (EDs) of a three-campus Academic Medical Center.

**Methods:**

A multi-phase prospective crossover trial was performed with the blood diversion device initially in use at one ED (Memorial) and standard equipment at the other ED (University) for 10 weeks. After a washout phase, a second 10-week study phase used the blood diversion device in the other ED (University) and standard equipment at the first ED (Memorial). Contaminants were identified by the clinical microbiology lab using standard criteria, and further defined by independent retrospective review by 3 infectious disease physicians prior to statistical analysis. An intention-to-treat analysis was performed, and Chi-square tests were used to compare contaminant rates among samples obtained using the blood diversion device versus standard equipment.

**Results:**

5,675 blood samples were obtained with 5,661 samples analyzed after 14 were deemed inconclusive by the ID physician review. There were 1,719 samples obtained at Memorial ED and 3,942 at University ED, with 2,836 samples collected during diversion device periods and 2,825 during standard equipment periods. Based on the ID physician review, the contaminant rates were 1.9% in diversion device periods versus 2.9% in standard equipment periods (P = 0.018). There was a marked difference in blood culture contamination rates between the two EDs with contaminant rates at the Memorial ED of 1.1% and 1.4% (P=0.57), and at the University ED of 2.3% and 3.5% (P=0.024) for the diversion device and standard equipment periods, respectively.

**Conclusion:**

The blood diversion device was able to significantly lower blood culture contamination rates overall by 1% at the institution’s two EDs (34% relative reduction), with a stronger effect noted at the campus with both a level 1 trauma center and transplant programs.

**Disclosures:**

**All Authors**: No reported disclosures

